# The chromosomal genome sequence of the upside-down jellyfish, *Cassiopea xamachana* Bigelow, 1892 (Rhizostomeae: Cassiopeidae) and an associated microbial metagenome bin

**DOI:** 10.12688/wellcomeopenres.27508.1

**Published:** 2026-09-15

**Authors:** Mónica Medina, Aki Ohdera, Anthony Bellantuono, Cheryl L. Ames, Allen Collins, William Fitt, Laura Miller, André Morandini, Jose Victor Lopez, Nina Pruzinsky, Graeme Oatley, Elizabeth Sinclair, Eerik Aunin, Noah Gettle, Camilla Santos, Michael Paulini, Haoyu Niu, Victoria McKenna, Rebecca O’Brien, Wellcome Sanger Institute Tree of Life Management, Samples and Laboratory team, Wellcome Sanger Institute Scientific Operations: Sequencing Operations, Wellcome Sanger Institute Tree of Life Core Informatics team, EBI Aquatic Symbiosis Genomics Data Portal Team, Aquatic Symbiosis Genomics Project Leadership

**Affiliations:** 1 Department of Ecology and Evolutionary Biology, University of California Los Angeles, Los Angeles, California, USA; 2 Stony Brook University Department of Ecology & Evolution, Stony Brook, New York, USA; 3 Graduate School of Agricultural Science, Tohoku University, Sendai, Japan; 4 National Museum of Natural History, Smithsonian Institution, Washington, District of Columbia, USA; 5 University of Georgia Odum School of Ecology, Athens, Georgia, USA; 6 Department of Mathematics, The University of Arizona, Tucson, Arizona, USA; 7 Zoology Department, Biosciences Institute, University of São Paulo, São Paulo, Brazil; 8 National Coral Reef Institute, Department of Biological Sciences, Nova Southeastern University, Fort Lauderdale, Florida, USA; 9 University Corporation for Atmospheric Research, Boulder, Colorado, USA; 10 NOAA Ocean Exploration Affiliate, Silver Spring, Maryland, USA; 11 Tree of Life Programme, Wellcome Sanger Institute, Hinxton, England, UK

**Keywords:** Cassiopea xamachana, cabbage-head jellyfish, genome sequence, chromosomal, Rhizostomeae, microbial metagenome assembly

## Abstract

We present a genome assembly from a specimen of *Cassiopea xamachana* (upside-down jellyfish; Cnidaria; Scyphozoa; Rhizostomeae; Cassiopeidae). The genome sequence has a total length of 326.12 megabases. Most of the assembly (98.3%) is scaffolded into 20 chromosomal pseudomolecules. The mitochondrial genome has also been assembled, with a length of 15.34 kilobases. From the metagenome data, we recovered one medium-quality bacterial bin assigned to *Endozoicomonas* sp.

## Species taxonomy

Eukaryota; Opisthokonta; Metazoa; Eumetazoa; Cnidaria; Scyphozoa; Rhizostomeae; Cassiopeidae; *Cassiopea*; *Cassiopea xamachana* Bigelow, 1892 (NCBI:txid12993).

## Background

*Cassiopea xamachana*, commonly known as the upside-down or mangrove jellyfish, was first described by Bigelow in 1892 from Jamaica. It is broadly distributed in Florida, the Bahamas, the Gulf of Mexico, and the Caribbean, although Holland *et al.* (2004) suggested that *C. xamachana* is synonymous with *C. andromeda* from the Red Sea. As its common name suggests, *C. xamachana* swimming medusae are epibenthic, and can reach sizes in the range 30–35 cm. Mature adults are characterised by colourful leaf-like appendages that extend upwards from highly ornamented oral arms and a concave bell with concentric blue and white patterns. These appendages can exhibit broad variation in colour, resulting from jellyfish-derived pigment. The eight oral arms extend beyond the bell margin and are lined with small secondary mouths surrounded by finger-like projections called digitata. These jellyfish lack a central mouth as medusae. The bell margin is marked by light and orientation-sensing structures called rhopalia. The life cycle of *C. xamachana*, as in many other rhizostome jellyfish, is metagenetic ( Schiariti *et al.*, 2024). The planula larva develops after gamete fertilisation, then settles and metamorphoses into sessile polyps. The polyps undergo transverse fission – known as strobilation – and parts of their bodies metamorphose into small jellyfish (ephyrae). The ephyrae then grow into what is typically recognised as the adult medusa stage.

*C. xamachana* medusae are epibenthic, found in shallow, low-flow areas around mangroves, often in seagrass beds. Thanks to their bell morphology and continuous pulsation, they remain resting on the seafloor. A distinguishing characteristic of the genus is the presence of photosynthetic dinoflagellate endosymbionts ( *Symbiodinium microadriaticum*) in the family Symbiodiniaceae ( Fitt & Trench, 1983). Resting in shallow environments allows *C. xamachana* to maximise exposure to sunlight, thereby supporting photosynthesis by the symbionts. *S. microadriaticum* provides *C. xamachana* with photosynthates in exchange for inorganic nutrients. The symbionts are housed in specialised cells called amoebocytes, which are found throughout the gelatinous layer, termed the mesoglea, which gives the animal tissue a light brown colouration ( Colley & Trench, 1983). The symbionts also play a critical role in the jellyfish life cycle, as the establishment of symbiosis is necessary for the polyps to undergo strobilation ( Hofmann *et al.*, 1996). The tight life-cycle association between the photosymbiont and the jellyfish has led to efforts to establish *Cassiopea* as a model system to study cnidarian-algal symbiosis-driven development ( Medina *et al.*, 2021; Ohdera *et al.*, 2018).

Researchers have also used *Cassiopea* to study the continuous fluid flow generated from their pulsing ( Hamlet & Miller, 2012; Santhanakrishnan *et al.*, 2012). Ecologists have studied the impact of *C. xamachana* on seagrass and other benthic flora ( Morejón-Arrojo *et al.*, 2025; Smulders *et al.*, 2023), and some have suggested that these jellyfish are ecosystem engineers ( Durieux *et al.*, 2023). Their pulsing is also an advantage in studying the evolution and molecular mechanisms of sleep behaviour, which was first described by Nath *et al.* (2017). The authors monitored their sleep–wake activity by enumerating changes in pulse frequency. Given the increasing utilisation of *C. xamachana* as a model system, new genomes provide the research community with additional resources to expand research potential. The first *Cassiopea* genome assembly is a scaffold-level assembly generated from a female clonal line ( Ohdera *et al.*, 2019). This sequencing effort provides the research community with a genome from a male individual and new comparative resources for future research.

## Methods

### Sample acquisition

An adult male medusa (specimen ID NSU0020601, ToLID jsCasXama1; Figure 1) was collected by snorkel in the vicinity of the Key Largo Marine Research Laboratory, Florida, USA (latitude 25.1015, longitude −80.4389), from 1–2 m depth in low-turbidity seawater over a sandy bottom. Another specimen was used for RNA sequencing (specimen ID NSU0020603, ToLID jsCasXama3). The specimens were collected under a valid Florida Saltwater Fishing License from an unrestricted area not within a protected park or sanctuary. Large adult conspecific medusae were abundant at the time of collection, though they have since become less common following the 2023 hyperthermal event in the Florida Keys.

**Figure 1.**
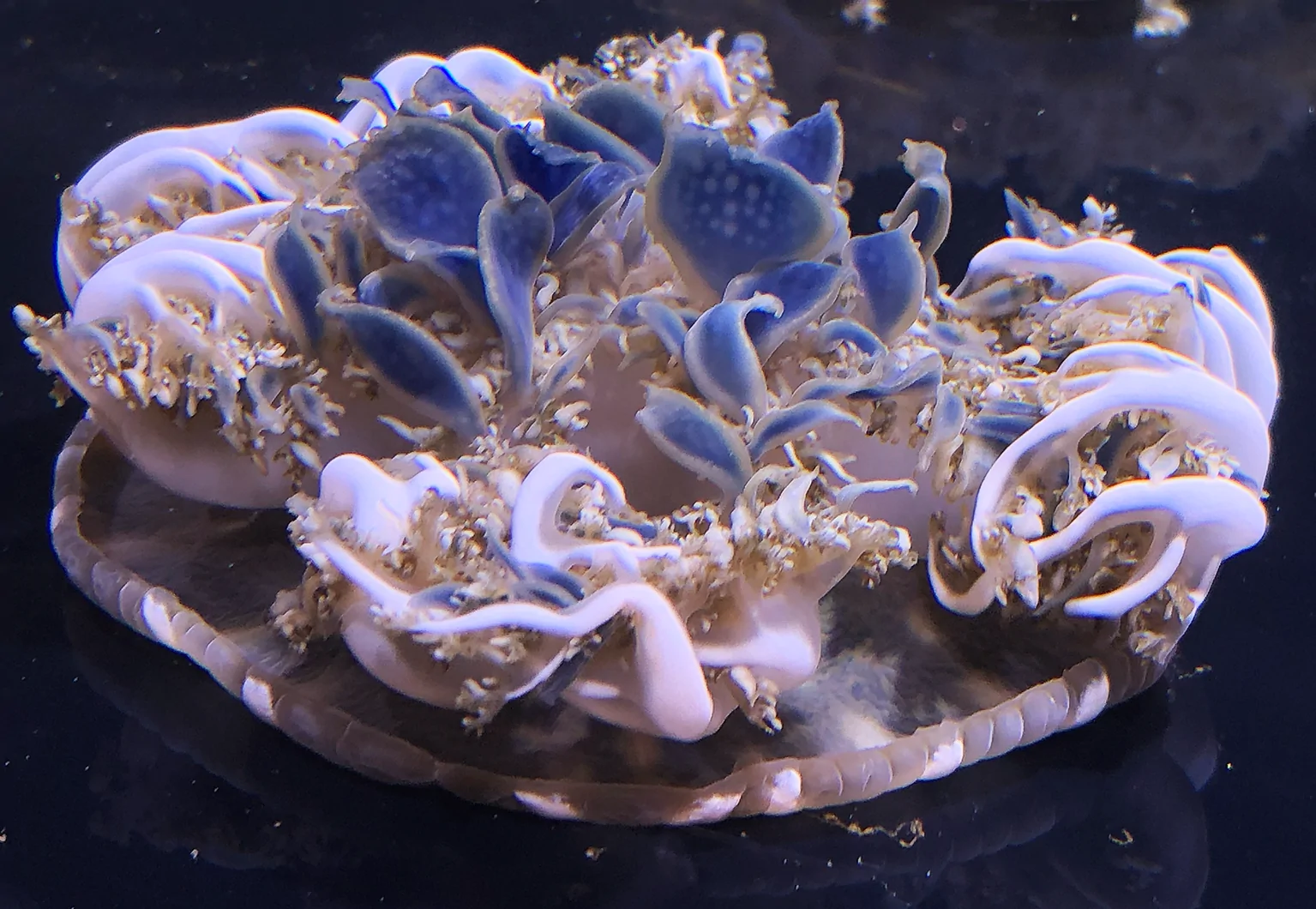
Photograph of the *C. xamachana* male sampled for sequencing.

### Nucleic acid extraction

Protocols for high molecular weight (HMW) DNA extraction developed at the Wellcome Sanger Institute (WSI) Tree of Life Core Laboratory are available on protocols.io ( Howard *et al.*, 2025). The jsCasXama1 sample was weighed and triaged to determine the appropriate extraction protocol. Tissue was homogenised by cryogenic disruption using the Covaris cryoPREP® Automated Dry Pulverizer. HMW DNA was extracted using the Manual MagAttract v3 protocol. We used centrifuge-mediated fragmentation to produce DNA fragments in the 8–10 kb range, following the Covaris g-TUBE protocol for ultra-low input (ULI). Sheared DNA was purified by automated SPRI (solid-phase reversible immobilisation). The concentration of the sheared and purified DNA was assessed using a Nanodrop spectrophotometer and Qubit Fluorometer using the Qubit dsDNA High Sensitivity Assay kit. Fragment size distribution was evaluated by running the sample on the FemtoPulse system. For this sample, the final post-shearing DNA had a Qubit concentration of 0.656 ng/μL, with a fragment size of 4.6 kb.

RNA was extracted from tissue of the jsCasXama3 specimen in the Tree of Life Laboratory at the WSI using the RNA Extraction: Automated MagMax™ *mir*Vana protocol. The RNA concentration was assessed using a Nanodrop spectrophotometer and a Qubit Fluorometer using the Qubit RNA Broad-Range Assay kit. Analysis of the integrity of the RNA was done using the Agilent RNA 6000 Pico Kit and Eukaryotic Total RNA assay.

### PacBio HiFi library preparation and sequencing

Library preparation and sequencing were performed at the WSI Scientific Operations core. Prior to library preparation, the DNA was fragmented to ~10 kb. Ultra-low-input (ULI) libraries were prepared using the PacBio SMRTbell® Express Template Prep Kit 2.0 and gDNA Sample Amplification Kit. Samples were normalised to 20 ng DNA. Single-strand overhang removal, DNA damage repair, and end-repair/A-tailing were performed according to the manufacturer’s instructions, followed by adapter ligation. A 0.85× pre-PCR clean-up was carried out with Promega ProNex beads.

The DNA was evenly divided into two aliquots for dual PCR (reactions A and B), both following the manufacturer’s protocol. A 0.85× post-PCR clean-up was performed with ProNex beads. DNA concentration was measured using a Qubit Fluorometer v4.0 (Thermo Fisher Scientific) with the Qubit HS Assay Kit, and fragment size was assessed on an Agilent Femto Pulse Automated Pulsed Field CE Instrument (Agilent Technologies) using the gDNA 55 kb BAC analysis kit. PCR reactions A and B were then pooled, ensuring a total mass of ≥500 ng in 47.4 μl.

The pooled sample underwent another round of DNA damage repair, end-repair/A-tailing, and hairpin adapter ligation. A 1× clean-up was performed with ProNex beads, followed by DNA quantification using the Qubit and fragment size analysis using the Agilent Femto Pulse. Size selection was performed on the Sage Sciences PippinHT system, with target fragment size determined by Femto Pulse analysis (typically 4–9 kb). Size-selected libraries were cleaned with 1.0× ProNex beads and normalised to 2 nM before sequencing.

The sample was sequenced on a Revio instrument (Pacific Biosciences, California, USA). Prepared libraries with a molarity greater than 2 nM were normalised to 2 nM, and 15 μL was used for making complexes. For libraries with a molarity below 2 nM, the available 10 μL library volume was used without normalisation. Primers were annealed and polymerases were bound to generate circularised complexes, following the manufacturer’s instructions. Complexes were purified using 1.2× SMRTbell beads, diluted to the Revio loading concentration of 200–300 pM, and spiked with a Revio sequencing internal control. The sample was sequenced on a Revio 25M SMRT cell. SMRT Link software (Pacific Biosciences), a web-based workflow manager, was used to configure and monitor the run and to carry out primary and secondary data analysis.

### Hi-C

***Sample preparation and crosslinking***

The Hi-C sample was prepared from 20–50 mg of frozen tissue of the jsCasXama1 sample using the Arima-HiC v2 kit (Arima Genomics). Following the manufacturer’s instructions, tissue was fixed and DNA crosslinked using TC buffer to a final formaldehyde concentration of 2%. The tissue was homogenised using the Diagnocine Power Masher-II. Crosslinked DNA was digested with a restriction enzyme master mix, biotinylated, and ligated. Clean-up was performed with SPRISelect beads before library preparation. DNA concentration was measured with the Qubit Fluorometer (Thermo Fisher Scientific) and Qubit HS Assay Kit. The biotinylation percentage was estimated using the Arima-HiC v2 QC beads.

***Hi-C library preparation and sequencing***

Biotinylated DNA constructs were fragmented using a Covaris E220 sonicator and size selected to 400–600 bp using SPRISelect beads. DNA was enriched with Arima-HiC v2 kit Enrichment beads. End repair, A-tailing, and adapter ligation were carried out with the NEBNext Ultra II DNA Library Prep Kit (New England Biolabs), following a modified protocol where library preparation occurs while DNA remains bound to the Enrichment beads. Library amplification was performed using KAPA HiFi HotStart mix and a custom Unique Dual Index (UDI) barcode set (Integrated DNA Technologies). Depending on sample concentration and biotinylation percentage determined at the crosslinking stage, libraries were amplified with 10–16 PCR cycles. Post-PCR clean-up was performed with SPRISelect beads. Libraries were quantified using the AccuClear Ultra High Sensitivity dsDNA Standards Assay Kit (Biotium) and a FLUOstar Omega plate reader (BMG Labtech).

Prior to sequencing, libraries were normalised to 10 ng/μL. Normalised libraries were quantified again to create equimolar and/or weighted 2.8 nM pools. Pool concentrations were checked using the Agilent 4200 TapeStation (Agilent) with High Sensitivity D500 reagents before sequencing. Sequencing was performed using paired-end 150 bp reads on the Illumina NovaSeq X.

### RNA library preparation and sequencing

Libraries were prepared using the NEBNext® Ultra II Directional RNA Library Prep Kit for Illumina (New England Biolabs), following the manufacturer’s instructions. Poly(A) mRNA was isolated from 100 ng of total RNA using oligo (dT) beads, converted to cDNA, and uniquely indexed; 14 PCR cycles were performed. Libraries were prepared with an intended fragment size of 100–300 bp. Libraries were quantified, normalised, and pooled equimolarly to a final concentration of 2.8 nM before dilution to 150 pM for loading. Sequencing was carried out on the Illumina NovaSeq X, generating paired-end reads.

### Genome assembly

Prior to assembly of the PacBio HiFi reads, a database of *k*-mer counts ( *k* = 31) was generated from the filtered reads using FastK. GenomeScope2 ( Ranallo-Benavidez *et al.*, 2020) was used to analyse the *k*-mer frequency distributions, providing estimates of genome size, heterozygosity, and repeat content.

The HiFi reads were assembled using Hifiasm ( Cheng *et al.*, 2021) with the --primary option. Haplotypic duplications were identified and removed using purge_dups ( Guan *et al.*, 2020). The Hi-C reads ( Rao *et al.*, 2014) were mapped to the primary contigs using bwa-mem2 ( Vasimuddin *et al.*, 2019), and the contigs were scaffolded in YaHS ( Zhou *et al.*, 2023) with the --break option for handling potential misassemblies. The scaffolded assemblies were evaluated using Gfastats ( Formenti *et al.*, 2022), BUSCO ( Manni *et al.*, 2021) and MerquryFK ( Rhie *et al.*, 2020).

The mitochondrial genome was assembled using MitoHiFi ( Uliano-Silva *et al.*, 2023). The MitoHiFi reference was the mitochondrial genome of *Spirostomum yagiui* ( NC_086648.1).

### Assembly curation

The assembly was decontaminated using the Assembly Screen for Cobionts and Contaminants ( ASCC) pipeline. TreeVal was used to generate the flat files and maps for use in curation. Manual curation was conducted primarily in PretextView and HiGlass ( Kerpedjiev *et al.*, 2018). Scaffolds were visually inspected and corrected as described by Howe *et al*. (2021). Manual corrections included 54 breaks, 112 joins, and removal of 41 haplotypic duplications. This reduced the scaffold count by 61.2%, increased the scaffold N50 by 1.5%, and reduced the total assembly length by 6.4%. The curation process is described at sanger-tol/curation-resources . PretextSnapshot was used to generate a Hi-C contact map of the final assembly.

### Assembly quality assessment

The Merqury.FK tool ( Rhie *et al.*, 2020) was run in a Singularity container ( Kurtzer *et al.*, 2017) to evaluate *k*-mer completeness and assembly quality for the primary assembly, the alternate haplotig set, and their union using the *k*-mer database ( *k* = 31) computed prior to genome assembly. The analysis outputs included assembly QV scores and completeness statistics.

The genome was analysed using the BlobToolKit pipeline, a Nextflow implementation of the earlier Snakemake version ( Challis *et al.*, 2020). PacBio reads were aligned to the assembly using minimap2 ( Li, 2018) and processed with SAMtools ( Danecek *et al.*, 2021) to generate coverage tracks. The pipeline runs BUSCO ( Manni *et al.*, 2021) using lineages identified from the NCBI Taxonomy ( Schoch *et al.*, 2020). For the three basal BUSCO lineages, eukaryota, bacteria and archaea, BUSCO genes are aligned to the UniProt Reference Proteomes database ( Bateman *et al.*, 2023) using DIAMOND BLASTp ( Buchfink *et al.*, 2021). The genome assembly is divided into chunks based on the density of BUSCO genes from the closest taxonomic lineage, and each chunk is aligned to the UniProt Reference Proteomes database using DIAMOND BLASTx. Sequences without DIAMOND BLASTx hits are extracted using seqtk and aligned to the NT database using BLASTn ( Altschul *et al.*, 1990). The outputs are consolidated into a BlobDir for visualisation in BlobToolKit. The BlobToolKit pipeline was developed using nf-core tooling ( Ewels *et al.*, 2020) and MultiQC ( Ewels *et al.*, 2016), with containerisation through Docker ( Merkel, 2014) and Singularity ( Kurtzer *et al.*, 2017).

### Metagenome assembly

The metagenome assembly was generated using MetaMDBG ( Benoit *et al.*, 2024) and binned using MetaBAT2 ( Kang *et al.*, 2019). The resulting bin set was optimised and refined using DAS Tool ( Sieber *et al.*, 2018). PROKKA ( Seemann, 2014) was used to identify tRNAs and rRNAs in each bin, CheckM ( Parks *et al.*, 2015) (checkM_DB release 2015-01-16) was used to assess bin completeness/contamination, and GTDB-Tk ( Chaumeil *et al.*, 2022) (GTDB release 214) was used to taxonomically classify bins. Taxonomic replicate bins were identified using dRep ( Olm *et al.*, 2017) with default settings (95% ANI threshold). All bins were assessed for quality and categorised as metagenome-assembled genomes (MAGs) if they met the following criteria: contamination ≤ 5%, presence of 5S, 16S, and 23S rRNA genes, at least 18 unique tRNAs, and either ≥ 90% completeness or ≥ 50% completeness with fully circularised chromosomes ( Bowers *et al.*, 2017). Bins that did not meet these thresholds, or were identified as taxonomic replicates of MAGs, were retained as ‘binned metagenomes’ provided they had ≥ 50% completeness and ≤ 10% contamination.

## Genome sequence report

### Sequence data

PacBio sequencing of the *Cassiopea xamachana* specimen generated 58.56 Gb (gigabases) from 10.22 million reads, which were used to assemble the genome. GenomeScope2.0 analysis using a *p* = 2 model gave a *k*-mer-based genome size estimate of 257.61 Mb, with a heterozygosity of 1.79% and repeat content of 19.77% ( Figure 2). These estimates guided expectations for the assembly. Based on the estimated genome size, the sequencing data provided approximately 189× coverage. Hi-C sequencing produced 444.64 Gb from 1 472.31 million reads, which were used to scaffold the assembly. RNA sequencing data were also generated and are available in public sequence repositories. Table 1 summarises the specimen and sequencing details.

**Figure 2.**
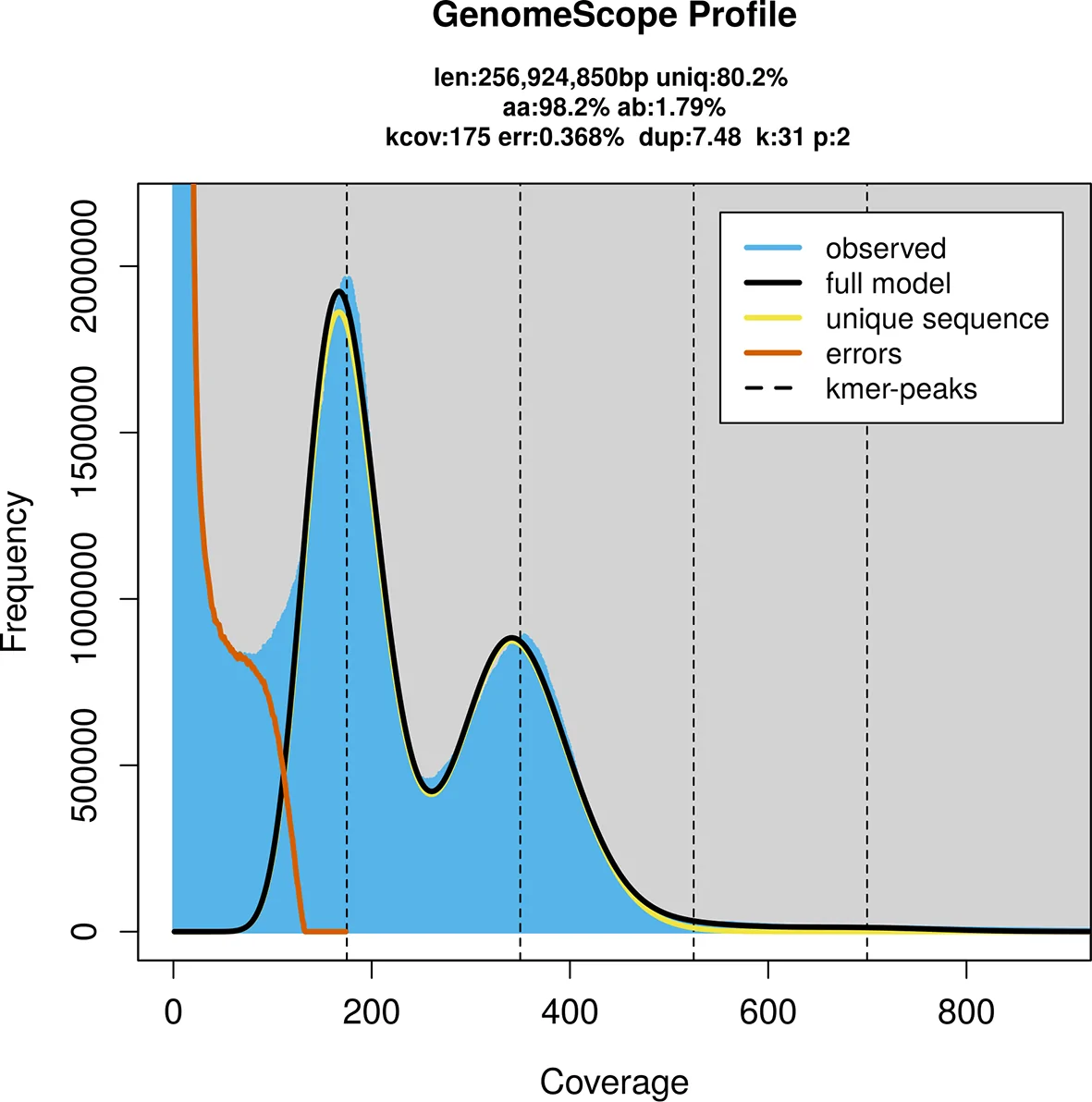
Frequency distribution of *k*-mers generated using GenomeScope2. The plot shows observed and modelled *k*-mer spectra, providing estimates of genome size, heterozygosity, and repeat content based on unassembled sequencing reads.

**Table 1.** Specimen and sequencing data for *Cassiopea xamachana* (BioProject PRJEB77019).

Platform	PacBio HiFi	Hi-C	RNA-seq
**ToLID**	jsCasXama1	jsCasXama1	jsCasXama3
**Specimen ID**	NSU0020601	NSU0020601	NSU0020603
**BioSample (source individual)**	SAMEA9196942	SAMEA9196942	SAMEA9196944
**BioSample (tissue)**	SAMEA9197060	SAMEA9197062	SAMEA9197018
**Tissue**	other somatic animal tissue	other somatic animal tissue	other somatic animal tissue
**Instrument**	Revio	Illumina NovaSeq X	Illumina NovaSeq X
**Run accessions**	ERR13332887	ERR13337206; ERR13337209; ERR13337205; ERR13363407	ERR13337208; ERR13337207
**Read count total**	10.22 million reads	1 472.31 million read pairs	59.89 million read pairs
**Base count total**	58.56 Gb	444.64 Gb	18.09 Gb

### Assembly statistics

The assembly strategy prioritised a complete and contiguous primary pseudohaplotype. Contigs corresponding to residual alternate haplotigs were also deposited in INSDC databases. The final primary assembly has a total length of 326.12 Mb in 561 scaffolds, with 2 934 gaps, and a scaffold N50 of 15.73 Mb ( Table 2).

**Table 2.** Genome assembly data for *Cassiopea xamachana.*

Genome assembly	Primary assembly
**Assembly name**	jsCasXama1.1
**Assembly accession**	GCA_964235115.1
**Alternate haplotype accession**	GCA_964235125.1
**Assembly level**	chromosome
**Span (Mb)**	326.12
**Number of chromosomes**	20
**Number of contigs**	3 495
**Contig N50**	0.14 Mb
**Number of scaffolds**	561
**Scaffold N50**	15.73 Mb
**Organelles**	Mitochondrial genome: 15.34 kb

The alternate haplotig set has a total length of 330.85 Mb in 12 986 contigs, with a contig N50 of 41.66 kb.

The scaffold count follows the NCBI assembly statistics and excludes organelle assemblies, which are reported separately where present.

Most of the assembly sequence (98.3%) was assigned to 20 chromosomal-level scaffolds. These chromosome-level scaffolds, confirmed by Hi-C data, are named according to size ( Figure 3; Table 3).

**Figure 3.**
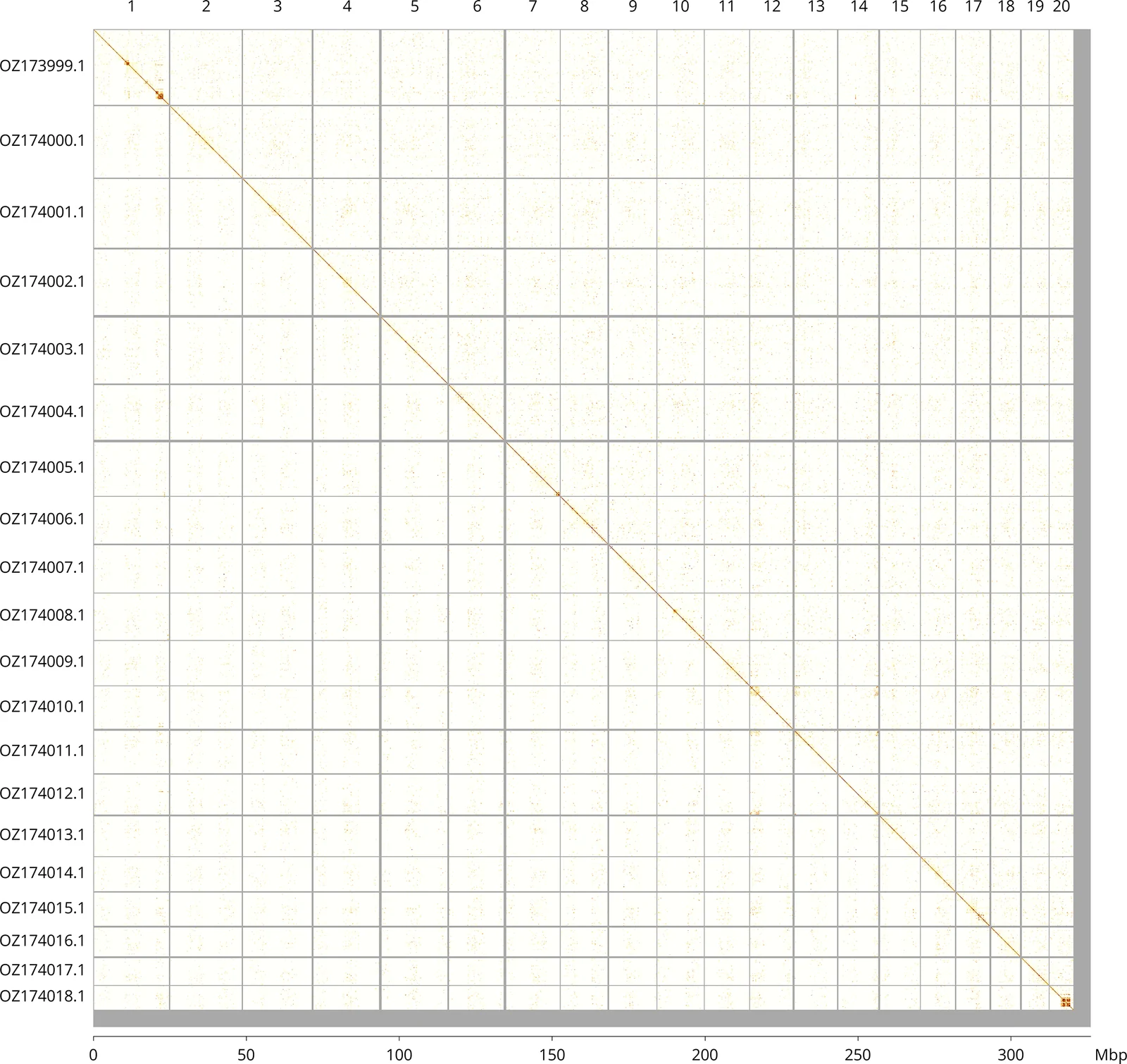
Hi-C contact map of the *Cassiopea xamachana* genome assembly. Assembled chromosomes are shown in order of size and labelled along the axes, with a megabase scale shown below. The plot was generated using PretextSnapshot.

**Table 3.** Chromosomal pseudomolecules in the primary genome assembly of *Cassiopea xamachana* jsCasXama1.

INSDC accession	Molecule	Length (Mb)	GC%
OZ173999.1	1	25	37
OZ174000.1	2	23.82	37
OZ174001.1	3	23.04	37
OZ174002.1	4	22.25	37
OZ174003.1	5	22.03	37
OZ174004.1	6	18.73	37
OZ174005.1	7	17.81	37
OZ174006.1	8	15.88	37
OZ174007.1	9	15.73	37
OZ174008.1	10	15.50	37
OZ174009.1	11	14.84	37
OZ174010.1	12	14.51	37.50
OZ174011.1	13	14.35	37
OZ174012.1	14	13.62	37
OZ174013.1	15	13.29	37
OZ174014.1	16	11.64	37
OZ174015.1	17	11.38	37
OZ174016.1	18	10.03	37
OZ174017.1	19	9.10	37
OZ174018.1	20	8.02	37

The mitochondrial genome was also assembled (length 15.34 kb, OZ174019.1). This sequence is included as a contig in the multifasta file of the genome submission and as a standalone record.

### Assembly quality metrics

The primary assembly achieves an estimated QV of 52.0 and a *k*-mer completeness of 71.96%. The union of the primary assembly and alternate haplotig set achieves an estimated QV of 53.1 and a *k*-mer completeness of 96.70% ( Figure 4). The alternate haplotig set has an estimated QV of 54.4 and a *k*-mer completeness of 63.25%.

**Figure 4.**
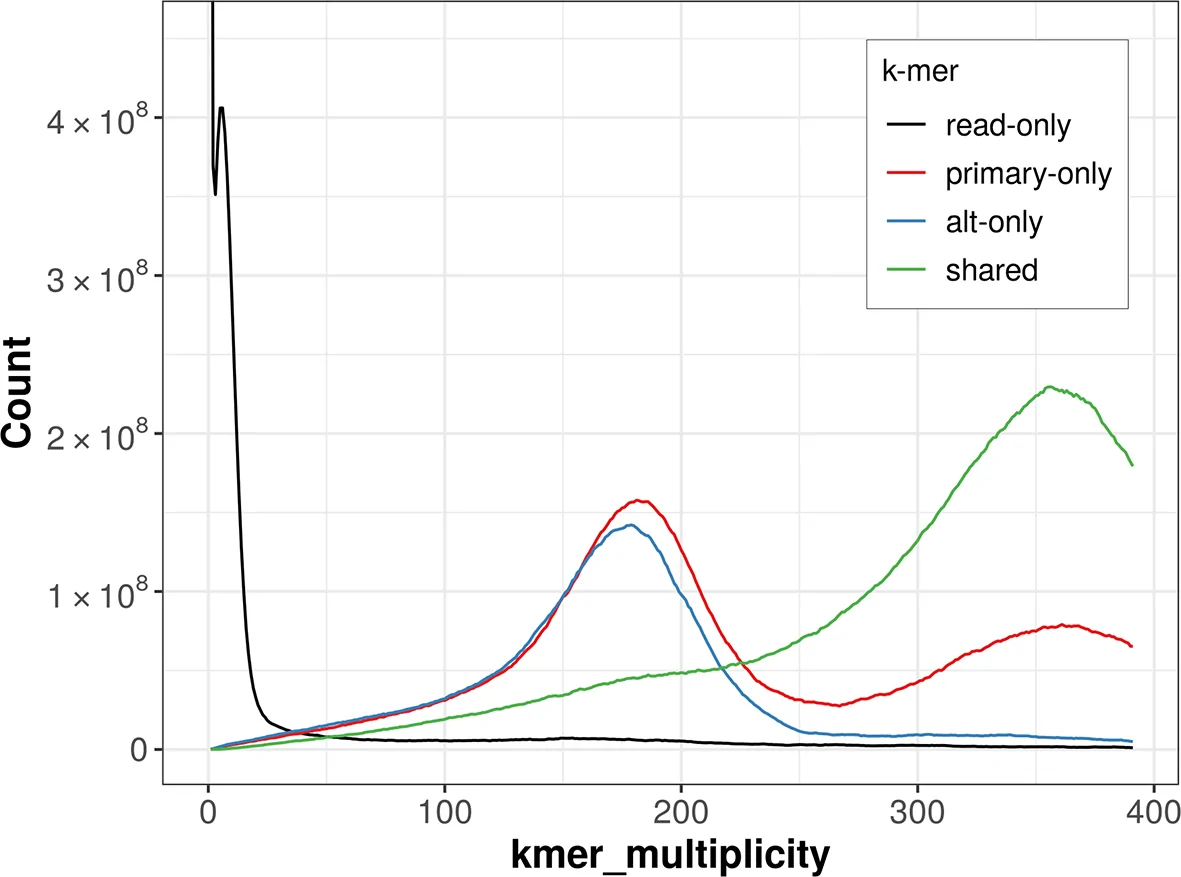
Evaluation of *k*-mer completeness using MerquryFK. This plot illustrates the recovery of *k*-mers from the original read data in the final assemblies. The horizontal axis represents *k*-mer multiplicity, and the vertical axis shows the number of *k*-mers. The black curve represents *k*-mers that appear in the reads but are not assembled. The green curve corresponds to *k*-mers shared by both assemblies, and the red and blue curves show *k*-mers found only in one assembly.

BUSCO v.5.5.0 analysis using the metazoa_odb10 reference set ( *n* = 954) identified 79.9% of the expected gene set (single = 79.6%, duplicated = 0.3%). The snail plot in Figure 5 summarises the scaffold length distribution and other assembly statistics for the primary assembly. The blob plot in Figure 6 shows the distribution of scaffolds by GC proportion and coverage.

**Figure 5.**
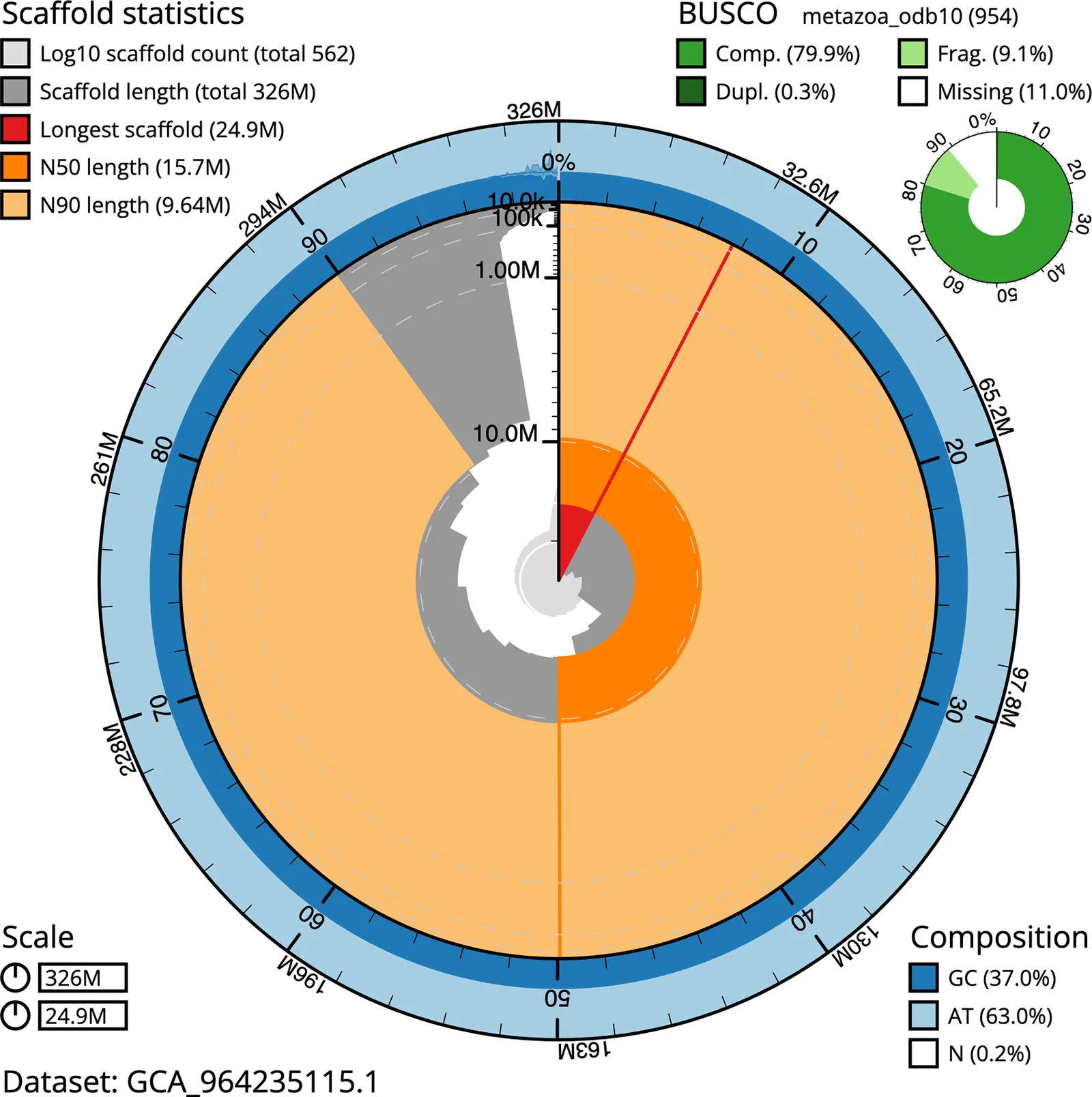
Assembly metrics for jsCasXama1.1. The BlobToolKit snail plot provides an overview of assembly metrics and BUSCO gene completeness. The circumference represents the length of the whole genome sequence, and the main plot is divided into 1 000 bins around the circumference. The outermost blue tracks display the distribution of GC, AT, and N percentages across the bins. Scaffolds are arranged clockwise from longest to shortest and are depicted in dark grey. The longest scaffold is indicated by the red arc, and the deeper orange and pale orange arcs represent the N50 and N90 lengths. A light grey spiral at the centre shows the cumulative scaffold count on a logarithmic scale. A summary of complete, fragmented, duplicated, and missing BUSCO genes in the metazoa_odb10 set is presented at the top right. An interactive version of this figure can be accessed on the BlobToolKit viewer.

**Figure 6.**
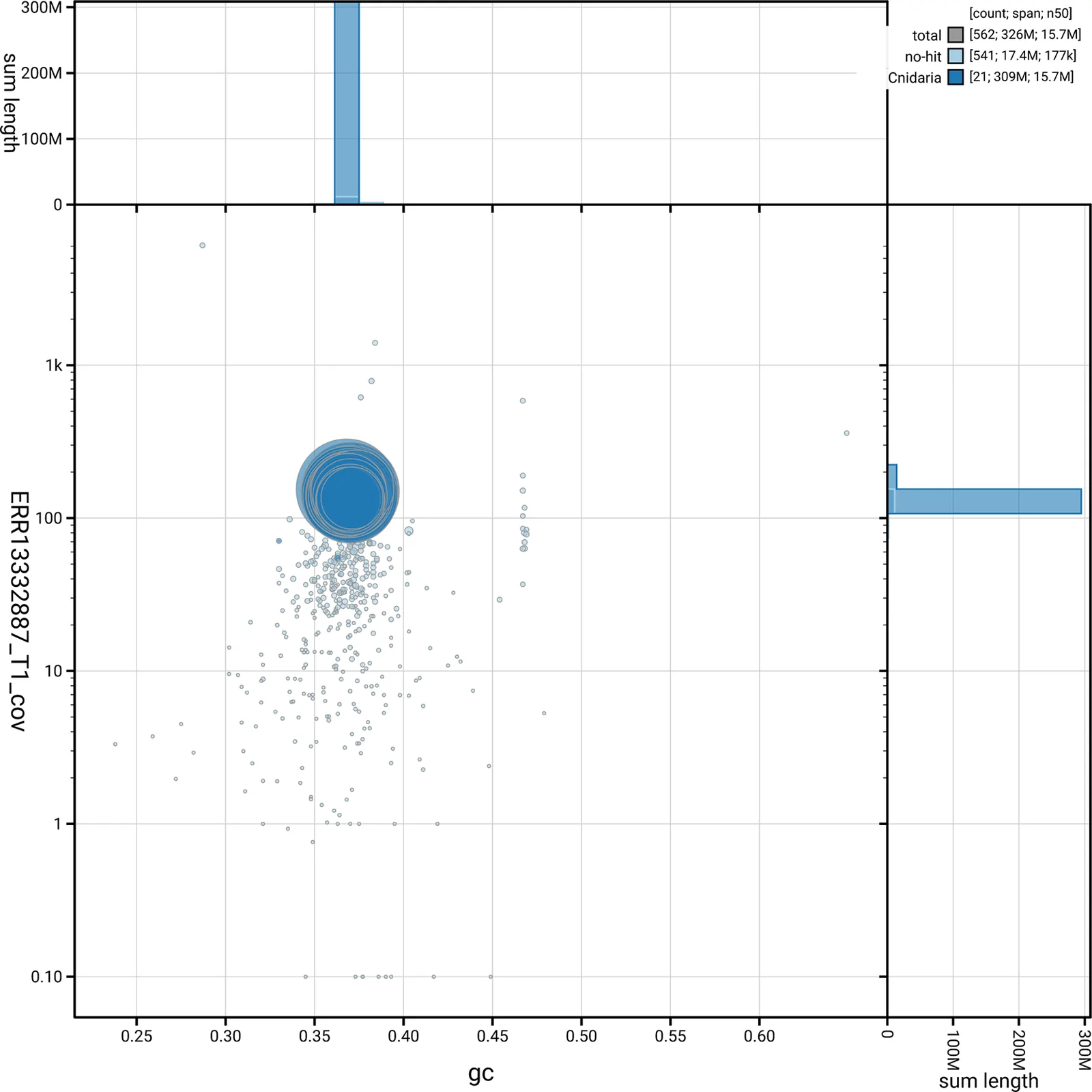
BlobToolKit blob plot for jsCasXama1.1. The plot shows base coverage (vertical axis) and GC content (horizontal axis). The circles represent scaffolds, with the size proportional to scaffold length and the colour representing phylum membership. The histograms along the axes display the total length of sequences distributed across different levels of coverage and GC content. An interactive version of this figure is available on the BlobToolKit viewer.

Table 4 lists the assembly metric benchmarks adapted from Rhie *et al.* (2021) and the Earth BioGenome Project Report on Assembly Standards January 2026. The EBP metric, calculated for primary assembly, is **5.C.Q52**.

**Table 4.** Earth Biogenome Project summary metrics for the *Cassiopea xamachana* assembly.

Measure	Value	Benchmark
EBP summary (primary)	5.C.Q52	6.C.Q40
Contig N50 length	0.14 Mb	≥ 0.1 Mb
Scaffold N50 length	15.73 Mb	= chromosome N50
Consensus quality (QV)	Primary: 52.0; alternate haplotig set: 54.4; primary plus alternate haplotig set: 53.1	≥ 40
*k*-mer completeness	Primary: 71.96%; primary plus alternate haplotig set: 96.70%; alternate haplotig set: 63.25%	> 90%
BUSCO	C:79.9% [S:79.6%, D:0.3%], F:9.1%, M:11.0%, n:954	S > 90%; D < 5%
Percentage of assembly assigned to chromosomes	98.30%	≥ 90%

*Notes:* The EBP summary uses log10(Contig N50); chromosome-level (C) or log10(Scaffold N50); Q (Merqury QV). BUSCO: C=complete; S=single-copy; D=duplicated; F=fragmented; M=missing; n=orthologues.

**Table 5.** Software versions and sources used for *Cassiopea xamachana.*

Software	Version	Source
BLAST	2.14.0	https://ftp.ncbi.nlm.nih.gov/blast/executables/blast+/
BlobToolKit	4.3.9	https://github.com/blobtoolkit/blobtoolkit
BUSCO	5.5.0	https://gitlab.com/ezlab/busco
bwa-mem2	2.2.1	https://github.com/bwa-mem2/bwa-mem2
CheckM	2015-01-16	https://ecogenomics.github.io/CheckM/
DAS Tool	1.1.2	https://github.com/cmks/DAS_Tool
DIAMOND	2.1.8	https://github.com/bbuchfink/diamond
dRep	3.4.0	https://github.com/MrOlm/drep
fasta_windows	0.2.4	https://github.com/tolkit/fasta_windows
FastK	1.1	https://github.com/thegenemyers/FASTK
GenomeScope2.0	2.0.1	https://github.com/tbenavi1/genomescope2.0
Gfastats	1.3.6	https://github.com/vgl-hub/gfastats
GTDB-Tk	1.2.1	https://github.com/Ecogenomics/GTDBTk
Hifiasm	0.19.8-r603	https://github.com/chhylp123/hifiasm
HiGlass	1.13.4	https://github.com/higlass/higlass
MerquryFK	1.1.0-c1	https://github.com/thegenemyers/MERQURY.FK
MetaBAT2	2.15-15-gd6ea400	https://bitbucket.org/berkeleylab/metabat
MetaMDBG	Pre-release	https://github.com/GaetanBenoitDev/metaMDBG
Minimap2	2.24-r1122	https://github.com/lh3/minimap2
MitoHiFi	3.2.2	https://github.com/marcelauliano/MitoHiFi
MultiQC	1.14; 1.17 and 1.18	https://github.com/MultiQC/MultiQC
Nextflow	23.10.0; 23.10.1	https://github.com/nextflow-io/nextflow
PretextSnapshot	0.0.4	https://github.com/sanger-tol/PretextSnapshot
PretextView	1.0.3	https://github.com/sanger-tol/PretextView
Prokka	1.14.5	https://github.com/tseemann/prokka
purge_dups	1.2.3	https://github.com/dfguan/purge_dups
samtools	1.17; 1.18; 1.19.2; 1.20; 1.6	https://github.com/samtools/samtools
sanger-tol/ascc	0.1.0	https://github.com/sanger-tol/ascc
sanger-tol/blobtoolkit	0.6.0	https://github.com/sanger-tol/blobtoolkit
sanger-tol/curationpretext	1.4.2	https://github.com/sanger-tol/curationpretext
Seqtk	1.4-r122	https://github.com/lh3/seqtk
Singularity	3.9.0	https://github.com/sylabs/singularity
TreeVal	1.1.0	https://github.com/sanger-tol/treeval
YaHS	1.1a.2	https://github.com/c-zhou/yahs

### Metagenome report

One bacterial bin was recovered from the metagenome assembly. It was assigned to *Endozoicomonas* sp. and was medium quality, with a size of 4.83 Mb, 160 contigs, 87.29% completeness and 2.49% contamination; it did not meet the criteria for a MAG.

## Author information

Contributors are listed at the following links:

• Members of the Wellcome Sanger Institute Tree of Life Management, Samples and Laboratory team
• Members of Wellcome Sanger Institute Scientific Operations – Sequencing Operations
• Members of the Wellcome Sanger Institute Tree of Life Core Informatics team
• Members of the EBI Aquatic Symbiosis Genomics Data Portal Team
• The Aquatic Symbiosis Genomics Project leadership

## Wellcome Sanger Institute – Legal and Governance

The materials that have contributed to this genome note have been supplied by a Tree of Life collaborator. The Wellcome Sanger Institute employs a process whereby due diligence is carried out proportionate to the nature of the materials themselves, and the circumstances under which they have been/are to be collected and provided for use. The purpose of this is to address and mitigate any potential legal and/or ethical implications of receipt and use of the materials as part of the research project, and to ensure that in doing so we align with best practice wherever possible.

The overarching areas of consideration are:

• Ethical review of provenance and sourcing of the material
• Legality of collection, transfer and use (national and international)

Each transfer of samples is undertaken according to a Research Collaboration Agreement or Material Transfer Agreement entered into by the Tree of Life collaborator, Genome Research Limited (operating as the Wellcome Sanger Institute) and in some circumstances other Tree of Life collaborators.

## Data Availability

European Nucleotide Archive: Cassiopea xamachana. Accession number PRJEB77019; https://identifiers.org/ena.embl/PRJEB77019. The genome sequence is released openly for reuse. The *Cassiopea xamachana* genome sequencing initiative is part of the Aquatic Symbiosis Genomics Project ( PRJEB43743) and the Sanger Institute Tree of Life Programme ( PRJEB43745). All raw sequence data and the assembly have been deposited in INSDC databases. The genome will be annotated using available RNA-Seq data and presented through the Ensembl pipeline at the European Bioinformatics Institute. Raw data and assembly accession identifiers are reported in Tables 1 and 2. Production code used in genome assembly at the WSI Tree of Life is available at https://github.com/sanger-tol . Table 5 lists software versions used in this study.
