# Supplementary material for: The chromosomal genome sequence of the upside-down jellyfish, Cassiopea xamachana Bigelow, 1892 (Rhizostomeae: Cassiopeidae) and an associated microbial metagenome bin
Source: Wellcome Open Res. 2026 Sep 15;11:671. [Version 1] doi: 10.12688/wellcomeopenres.27508.1 (PMC13736059; doi:10.12688/wellcomeopenres.27508.1)
Supplement: Peer review history

# Reviewer report: Reviewer response for version 1

**DOI:** 10.21956/wellcomeopenres.30277.r172013

**Published:** 2026-11-04

**Authors:** Adrián Jaimes-Becerra

**Affiliations:** 1 Tohoku University, Sendai, Japan

This Data Note presents a chromosome-scale reference genome for a male *Cassiopea xamachana*. The assignment of 98.3% of the assembled sequence to 20 chromosomal pseudomolecules makes this a valuable resource for comparative genomics. Could the authors clarify how they calculated the reported 189× sequencing coverage? The 58.56 Gb of HiFi data gives about 227× coverage using the estimated genome size (257.61 Mb) or 180× using the assembly size (326.12 Mb). Please specify which values were used and briefly discuss why the assembly is about 27% larger than the genome size estimate.

Are sufficient details of methods and materials provided to allow replication by others?

Yes

Is the rationale for creating the dataset(s) clearly described?

Yes

Are the datasets clearly presented in a useable and accessible format?

Yes

Are the protocols appropriate and is the work technically sound?

Yes

Reviewer Expertise:

Evolutionary biology, Cnidaria genomics, venom evolution

I confirm that I have read this submission and believe that I have an appropriate level of expertise to confirm that it is of an acceptable scientific standard.

# Reviewer report: Reviewer response for version 1

**DOI:** 10.21956/wellcomeopenres.30277.r169419

**Published:** 2026-10-27

**Authors:** Bingpeng Xing

**Affiliations:** 1 Third Institute of Oceanography Ministry of Natural Resources, Xiamen, Fujian, China

The main text reports a GenomeScope estimated value of 257.61 Mb, whereas Figure 2 shows 256,924,850 bp; the final assembly length is 326.12 Mb, which is significantly higher than the aforementioned estimate, and its possible reasons should be discussed. In addition, 58.56 Gb divided by 257.61 Mb is approximately 227×, which does not yield the 189× stated in the text. If 189× is calculated based on filtered or host-derived valid data volume, the corresponding data volume and calculation method should be clearly stated. It is recommended to report the read counts, base counts, and the proportion of host reads both before and after filtering.

 Please provide the collection date, specific tissue sampled, storage and transportation conditions, the basis for male identification, and the sex of the individual used for RNA sequencing. Given the taxonomic controversy of this group mentioned in the background, the basis for species identification and the preservation status of voucher specimens should also be described. In Table 5, "2015-01-16" for CheckM appears to be the database release date and should be separated from the software version; labeling MetaMDBG only as "Pre-release" is insufficient for reproducing the analysis, and providing a specific version or commit ID is recommended.

Are sufficient details of methods and materials provided to allow replication by others?

Partly

Is the rationale for creating the dataset(s) clearly described?

Yes

Are the datasets clearly presented in a useable and accessible format?

Yes

Are the protocols appropriate and is the work technically sound?

Partly

Reviewer Expertise:

marine biology， genome，mangrove，marine biodiversity

I confirm that I have read this submission and believe that I have an appropriate level of expertise to confirm that it is of an acceptable scientific standard, however I have significant reservations, as outlined above.

# Reviewer report: Reviewer response for version 1

**DOI:** 10.21956/wellcomeopenres.30277.r169414

**Published:** 2026-10-13

**Authors:** Wai Lok So

**Affiliations:** 1 The Hang Seng University of Hong Kong (Ringgold ID: 272025), Hong Kong, Hong Kong

The manuscript presents a male genome assembly of the upside-down jellyfish, *Cassiopea xamachana. *The background was well written, with sufficient details concerning the biology of *C. xamachana*. 

 Some comments regarding the methods and assembly:

**Methods**

 1. How the authors confirm the collected sample is a male? Please kindly state the methods for identification.

 2. Also state the sex of the sample that was used for RNA sequencing.

 3. Were the samples collected in the field stored in liquid nitrogen immediately or kept alive until subsequent experiments? Please kindly state.

**BUSCO**

 The BUSCO value of the assembly is low, considering it is around 79.9% being annotated. Please address this issue.

**Figure**

 1. A scale bar should be included.

 2. The sample shown in the figure 1 is for genome/RNA sequencing? Please clarify the information in the legend.

Are sufficient details of methods and materials provided to allow replication by others?

Partly

Is the rationale for creating the dataset(s) clearly described?

Yes

Are the datasets clearly presented in a useable and accessible format?

Yes

Are the protocols appropriate and is the work technically sound?

Partly

Reviewer Expertise:

Evolution, genomics, invertebrate biology

I confirm that I have read this submission and believe that I have an appropriate level of expertise to confirm that it is of an acceptable scientific standard, however I have significant reservations, as outlined above.

# Reviewer report: Reviewer response for version 1

**DOI:** 10.21956/wellcomeopenres.30277.r172008

**Published:** 2026-10-10

**Authors:** Abdelmalek Lekired

**Affiliations:** 1 University of New Mexico, Albuquerque, New Mexico, USA

The chromosomal genome sequence of the upsidedown jellyfish, Cassiopea xamachana Bigelow, 1892 (Rhizostomeae: Cassiopeidae) and an associated microbial metagenome bin

 The authors state that the assembly was performed using Hifiasm, but the final primary assembly is reported as 561 scaffolds containing 2,934 gaps. Since Hifiasm primarily generates contigs, please clarify whether these scaffolds and gaps were generated during the subsequent Hi-C/YaHS scaffolding step. The authors should clearly distinguish the statistics of the Hifiasm primary contig assembly from those of the final scaffolded assembly.

 The head rows of the table two are not clear ? Genome assembly and Primary assembly ??

 The Table 3 showed the length of each pseudochromosome, however, the sum of them is 320.57 Mb but you mentioned in the Table (2) 326.12 Mb ??

 The BUSCO is too low “79%” for a chromosome level assembly ! Please provide the BUSCO metrics for the hifiasm primary assembly.

Are sufficient details of methods and materials provided to allow replication by others?

Partly

Is the rationale for creating the dataset(s) clearly described?

Yes

Are the datasets clearly presented in a useable and accessible format?

Yes

Are the protocols appropriate and is the work technically sound?

Partly

Reviewer Expertise:

Bacterial, Fungal, and invertebrate genome assembly and annotation

I confirm that I have read this submission and believe that I have an appropriate level of expertise to confirm that it is of an acceptable scientific standard, however I have significant reservations, as outlined above.
